# Medical Topography of Nottinghamshire

**Published:** 1813-08

**Authors:** 


*To the Editors of the Medical and Physical Journal.*

GENTLEMEN,

PERFECTLY coinciding in your opinion on the variously-useful information to the profession and to the public, likely to be obtained by a medical view of the different counties of the United Kingdoms, I now endeavor to contribute my mite to the general stock, by transmitting a topographico-medical survey of the county of Nottingham.

I trust you will excuse me suggesting to your correspondents the additional advantages deducible from an enumeration of the inaugural theses of the different physicians, and from a notice of whatever essays or memoirs may have been given to the world by the different medical characters of each county, whether diffused in the extensively-circulated monthly journals, or in separate volumes. This may be accomplished without any considerable trouble.

Allow me also the liberty of remarking, that no very essential benefits can result from publishing these surveys in alphabetical order, which may have the effect of preventing for a length of time the appearance of many of the different counties, and altogether of retarding the views which I am disposed to entertain on this subject. I would recommend that the topographical surveys should be published in each Number as they are received by the respectable Editors of the the Medical Journal; and when they have been given to the medical world in this detached manner, I should have great pleasure in seeing them collected in regular alphabetical order in one number of the same work. This number will always prove a valuable source of reference, and our brethren with a very inconsiderable degree of trouble may be acquainted with the names, residence, and different departments, of all the medical men in the United Kingdoms, with the inaugural theses of graduates, and the publications of every practitioner. Proceeding now to the topographical survey of Nottinghamshire, I remain, Gentlemen,

Your constant reader, and occasional contributor,

H. B.

In the neighbourhood of Nottingham is a large handsome Infirmary, supported by voluntary subscription, and gratuitously attended by the medical officers. The number of patients admitted into this Infirmary during the last year (1812) amounted to 45, and the out-patients to 336. This Infirmary has received patients during the last 31 years, and has admitted and discharged in that period, in and out patients, to the number of 37,161. Of this number 4107 persons were admitted on sudden accidents; and there have been, since its first opening, 190 amputations, 9 trepanned, and 51 cut for the stone. The medical officers of this excellent institution are the following:—Dr. John Storer, consulting phys. extra. for life; Dr. William Marsden, Dr. Charles Pennington, Dr. Alexander Manson, physicians; Mr. Thomas Wright, Mr. John Attenburrow, and Mr. John Wright, surgeons; Mr. Robert Thompson, house-surgeon, apothecary, and secretary; Mr. Carden Thompson, apprentice in the hospital.

A handsome and extremely commodious building has lately been erected in the neighbourhood of Nottingham for the reception of lunatics; the medical officers of which are:—Dr. John Storer, consulting physician; Dr. Charles Pennington, physician; Mr. Henry Oldknow, surgeon; Mr. Morris, director.

Physicians practising and residing in the town, with their inaugural theses, &c.

Dr. John Storer,* (*De Anginâ Malignâ,*) Glasg. 1771.

Dr. William Marsden, (*De Variolis,*) Edinb. 1792.

Dr. C. Pennington, (*De Puerperarum Febre,*) Glasg. 1795.

Dr. H. Payne, (*De Antimonio,*) Edinb. 1810.

Dr. Alex. Manson, (*De Synochâ,*) Edinb. 1811.

---

* Dr. Storer has given an instructive paper in the third volume of the Medical and Chirurgical Transactions, intitled, "An Instance of the entire Want of Pulsation in the Arteries of Paralytic Limbs."

Surgeons practising and residing in the town of NOTTINGHAM:—Mr. Thomas Wright, Mr. Attenburrow, Mr. John Wright, Mr. Maddox, Mr. Basnett, Mr. Butlin, Mr. Williams, Mr. Calvert, Mr. Buck, Mr. Watts, Mr. Stanley, Mr. Higginbottom, Mr. Allen, Mr. Oldknow.*

NEWARK.—The following medical gentlemen practise and reside in Newark:—Physician, Dr. Robert Buck, (*De Ischuria Renali,*) Edinb. 1778.—Surgeons, Mr. Lacey, Mr. Parker, Messrs. Bland and Deeping, Mr. Ashwell, Mr. Thompson, Mr. Cooke.

RETFORD.—Physician, Dr. John Bigsby.—Surgeons, Messrs. Cavie and Holmes, Messrs. Russel and Hartshorn, Mr. Flower, Mr. Ince.

SOUTHWELL.—Mr. Thomas Falkner, Mr. Hutchinson,† Mr. Cooke.

MANSFIELD.—Mr. Paulson, Mr. Savage, Mr. Bowmer.

TUXFORD.—Mr. Clarke.

DUNHAM.—Mr. Eyre.

SUTTON-ON-TRENT.—Mr. Spry, Mr. Mann.

SUTTON-IN-ASHFIELD.—Mr. Bacheler.

WARSOP.—Mr. Robinson.

ARNOLD.—Mr. Keyworth.

CARLTON.—Mr. Mann.

BINGHAM.—Mr. Rose.

CAR-COULSTON.—Mr. Blagg.

ALLERTON.—Mr. Ward.

WORKSOP.—Mr. Marson,‡ Mr. Clarke.

---

*To the Editors of the Medical and Physical Journal.*

GENTLEMEN,

ON looking over your last Number, (for July,) I find there a case of obstinate *vomiting* related by Dr. Yeats; in which, after having used many ineffectual remedies, he at length discovered the utility of mercury, in arresting the progress of so obstinate a disease. As a farther proof of the success of this practice, when properly directed,

* Mr. Oldknow has given two ingenious papers in the volumes of the Edinburgh Med. and Surg. Journal on Hydrophobia, and on the operation of tying the great Saphena Vein.

† Mr. Hutchinson is the author of the Biographia Medica, and of several papers in the Memoirs of the Medical Society of London, and in the London and Edinburgh Medical Journals, &c.

‡ Mr. Marson is the author of one or two papers on the subject of Vaccination, in one of the early volumes of this Journal.

